# Unveiling the Mysteries of CLEC3B: Physiological Roles, Pathological Impacts, and Research Gaps

**DOI:** 10.3390/cells15131160

**Published:** 2026-06-25

**Authors:** Le Li, Liang Guo

**Affiliations:** 1 Key Laboratory of Exercise and Health Sciences of the Ministry of Education, Shanghai University of Sport, Shanghai 200438, China; 2421526023@sus.edu.cn; 2 School of Exercise and Health, Collaborative Innovation Center for Sports and Public Health Shanghai University of Sport, Shanghai 200438, China; 3 Shanghai Key Lab of Human Performance, Shanghai University of Sport, Shanghai 200438, China; 4 Shanghai Frontiers Science Research Base of Exercise and Metabolic Health, Shanghai University of Sport, Shanghai 200438, China

**Keywords:** C-type lectin-like domain, tetranectin, CLEC3B, matrix-associated matricellular protein, inflammation, cardiovascular disease, cancer, osteoarthritis, biomarker, extracellular matrix remodeling

## Abstract

*CLEC3B* (C-type lectin domain family 3 member B), also known as tetranectin (TN), is a secreted trimeric protein containing a C-type lectin-like domain (CTLD). Located on chromosome 3p21.31. CLEC3B maintains organismal homeostasis through roles in immune regulation, angiogenesis, and musculoskeletal biology. Genetic studies demonstrate that CLEC3B deficiency impairs tissue repair, bone mineralization, and fibrinolytic balance. Altered CLEC3B expression is linked to cardiovascular disease progression, autoimmune susceptibility, and cancer prognosis. This review synthesizes CLEC3B’s biological functions and evaluates its translational potential: circulating CLEC3B as a prognostic and diagnostic biomarker; tissue-resident CLEC3B as a predictive marker for therapeutic response; and CLEC3B-related pathways as candidate therapeutic targets for potential amenable to replacement or inhibition strategies. We identify critical research gaps to guide future investigations, including limited structural data, ambiguous glycan specificity, incomplete proteolytic network mapping, and lack of validated disease models. Collectively, these gaps currently preclude definitive therapeutic claims.

## 1. An Overview of CLEC3B and Its Family

C-type lectin domain family 3 member B (CLEC3B, with the encoding gene *CLEC3B* in humans and *Clec3b* in mice), also known as tetranectin (TN), is a secreted homotrimeric protein containing a C-type lectin-like domain (CTLD) [1]. Unlike canonical C-type lectins, the CLEC3B CTLD does not mediate Ca^2+^-dependent carbohydrate recognition, although it retains calcium-binding sites that contribute to structural stability and protein–protein interactions. While CLEC3B is classified within the C-type lectin domain superfamily based on sequence and structural homology, its CTLD lacks canonical Ca^2+^-binding motifs (e.g., EPN/WND) required for carbohydrate recognition. Nevertheless, CLEC3B retains non-canonical Ca^2+^-binding sites that contribute to structural stability and protein–protein interactions, distinguishing it from classical C-type lectins that function as calcium-dependent carbohydrate-binding proteins (lectins). C-type like domain is involved in intercellular adhesion and immune response processes. CLEC3B is a secreted protein synthesized with an N-terminal signal peptide (residues 1–22) that directs its translocation into the endoplasmic reticulum and subsequent trafficking through the Golgi apparatus for classical secretion. Once secreted, CLEC3B accumulates in the ECM and blood plasma [2]. In addition, CLEC3B has been detected in exosomes, though the precise mechanism of its exosomal sorting remains incompletely characterized. Proteomic analyses demonstrated that exosomal CLEC3B could regulate AMPK–VEGF signaling in recipient endothelial cells [3]. In addition, CLEC3B can be detected in serum, stroma, and tumor cells of patients with different tumor types [4]. CLEC3B is structurally homologous to the C-type lectin superfamily but functionally distinct from canonical lectins, occupying an intermediate position that justifies its CTLD-containing designation without implying bona fide lectin activity.

The pleiotropic functions of CLEC3B span tissue repair, skeletal homeostasis, metabolic regulation, immune modulation, and neuronal survival, with its dysregulation linked to cardiovascular disease, cancer, neurodegeneration, and musculoskeletal disorders [5]. Rather than providing an exhaustive preview, the present review is organized to systematically dissect these functions: Section 3 examines the molecular mechanisms underlying CLEC3B’s physiological roles [6], Section 4 explores its disease-specific pathophysiology and context-dependent functional duality [7], and Section 5 andSection 6 evaluate translational applications and identify critical research gaps [8]. To frame these discussions, we adopt the FDA-NIH BEST Resource definitions for biomarkers [9] and the three-criterion framework (dysregulation, mechanistic causality, druggability) for therapeutic target validation [10], as detailed below [11]. 

For this review, we adopt the FDA-NIH BEST definition of a biomarker as “a defined characteristic measured as an indicator of biological or pathogenic processes, or responses to intervention” [12,13], encompassing diagnostic, prognostic, and predictive applications (e.g., plasma CLEC3B for coronary artery diagnosis, low CLEC3B for hepatocellular carcinoma prognosis, and CLEC3B levels for lung cancer therapy stratification). A novel therapeutic target is defined as a molecular entity whose modulation confers therapeutic benefit [14], requiring disease relevance, mechanistic causality, and druggability. As applied to CLEC3B, disease relevance is supported by multiple association studies, whereas mechanistic causality and druggability remain primarily preclinical.

## 2. Structure of CLEC3B and Its Molecular Features

**Figure 1:**
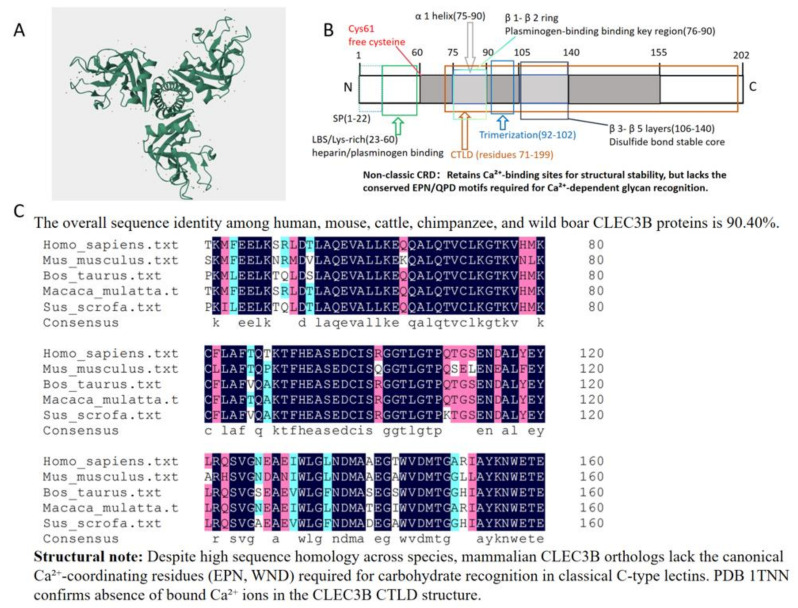
Molecular features of CLEC3B. (**A**) View the UniProt features on this structure of the CLEC3B protein in the feature viewer. (**B**) Schematic diagram illustrates the structure of human CLEC3B protein from the N-terminus to the C-terminus. The diagram highlights the signal peptide (SP, residues 1–22); the lysine-rich region (residues 23–60) containing the lysine-binding site (LBS) for heparin and plasminogen binding; a free cysteine (Cys61); the α1-helix (75–90) within the plasminogen-binding key region (76–90); the trimerization α-helix (residues 92–102); the β3–β5 layers (106–140) forming the disulfide bond stable core; and a C-type lectin-like domain (CTLD, 71–199) (https://www.uniprot.org/uniprotkb/P05452/entry#function, accessed on 24 June 2024). Notably, although CLEC3B contains a CTLD, it lacks a functional carbohydrate recognition domain (CRD) and does not mediate Ca^2+^-dependent glycan recognition, however, it retains Ca^2+^-binding sites that contribute to structural stability and protein–protein interactions. (**C**) Comparison and alignment of CLEC3B protein sequences of *Homo sapiens*, *Mus musculus*, *Bos taurus*, *Macaca mulatta*, and *Sus scrofa*. UniProt accession number: P05452 (accessed on [24 June 2024]). The subcellular journey of CLEC3B begins with co-translational import into the ER via its N-terminal signal peptide, followed by Golgi-mediated processing and secretion. Notably, CLEC3B lacks a transmembrane domain or GPI-anchor signal, suggesting that its presence in exosomes likely results from cargo sorting into multivesicular bodies (MVBs) rather than membrane-anchored shedding. Recent exosome proteomics studies have identified CLEC3B in vesicles from diverse cell types, including hepatocytes and cancer cells [29], but the specific sorting machinery (e.g., ESCRT-dependent vs. tetraspanin-enriched microdomain pathways) has not been defined. Functionally, soluble CLEC3B in plasma and ECM appears to act as a broad-spectrum regulator of fibrinolysis and matrix remodeling, whereas exosomal CLEC3B may provide targeted, localized delivery to specific cell populations—such as the observed transfer of tumor-derived exosomal CLEC3B to endothelial cells to modulate angiogenesis [2]. This dual distribution pattern (diffuse ECM/plasma vs. packaged exosomal) raises the possibility of context-dependent functional specialization, though direct comparative studies of soluble versus exosome-associated CLEC3B activity are currently lacking.

C-type lectins (CTLs) are Ca^2+^-dependent carbohydrate-binding proteins characterized by functional carbohydrate recognition domains (CRDs)—structural subdomains containing conserved Ca^2+^-binding motifs (EPN/WND) that mediate sugar recognition. C-type lectin-like domains (CTLDs) share the tertiary fold of CRDs but lack canonical Ca^2+^-binding motifs and may not bind carbohydrates. CLEC3B is not a canonical CTL; its C-terminal CTLD (residues 71–199) adopts the CTLD fold with distant structural homology to CRDs, yet it lacks canonical Ca^2+^-binding motifs (e.g., EPN/WND) required for Ca^2+^-dependent glycan binding [15]. Consequently, CLEC3B functions as a C-type lectin-like protein that mediates protein–protein interactions (e.g., with plasminogen, tPA, fibronectin) rather than Ca^2+^-dependent glycan binding. This distinction is critical because the CTLD nomenclature is based on structural fold conservation not on functional equivalence with canonical CRDs. The schematic representation of the CLEC3B protein structure is shown in Figure 1A. CLEC3B protein forms a homotrimer of three identical, non-covalently linked subunits (≈20 kDa each) [16,17]. Each mature monomer (181 amino acids) folds into three distinct domains [18]. The N-terminal domain is a lysine-rich one and mediates heparin binding [19]. This region plays a key role in the interaction of CLEC3B with ECM components and cell-surface receptors, such as integrins, thereby participating in the regulation of important biological processes, including cell adhesion, migration, and tissue remodeling [20]. This region contains the core structure responsible for CLEC3B forming homotrimers, namely a stable triple α-helical coiled-coil structure that tightly assembles the three subunits through stable interhelical interactions. This unique coiled-coil structure endows CLEC3B with exceptional structural stability and biological activity [20]. The C-terminal CTLD of CLEC3B (residues 71–199) adopts the characteristic β-sandwich fold of the C-type lectin-like domain family, consisting of two antiparallel β-sheets and one C-terminal α-helix (Figure 1B). As noted above, this CTLD lacks canonical Ca^2+^-binding motifs and therefore does not mediate Ca^2+^-dependent carbohydrate recognition; instead, its CTLD mediates Ca^2+^-independent protein–protein interactions with ligands such as plasminogen and tPA [20]. The C-terminal CTLD of CLEC3B adopts a β-sandwich fold structurally related to the C-type lectin domain family, consisting of two antiparallel β-sheets and one C-terminal α-helix (Figure 1B) [21]. This fold exhibits distant structural similarity to the fibrinogen-related domain (FReD)—a separate protein domain family found in ficolins, tenascins, and tachylectins. However, despite this structural resemblance, CLEC3B is definitively annotated as a CTLD-containing protein, not as an FReD-containing protein. This classification is supported by structural data (PDB: 1HTN [trimeric full-length structure, 2.8 Å] and 1TN3 [isolated CRD structure, 2.0 Å] and UniProt annotation (P05452) [22]. In the PDB entry 1HTN, the β-strands of this CTLD are annotated as β1 (residues 19–22), β2 (residues 27–30), β3 (residues 35–38), β4 (residues 43–46), β5 (residues 55–58), and β6 (residues 63–66), while the α-helix is annotated as α1 (residues 72–82). These secondary structural elements form the basis for constructing its more complex supersecondary structures and functions [21]. Structurally, the two antiparallel β-sheets (β1-β3 and β4-β6) create a compact β-sandwich scaffold; the C-terminal α1-helix is positioned near the inter-subunit interface, potentially contributing to homotrimer stabilization. The β-sheet surface, particularly the region encompassing β2–β3 (residues 27–38 [PDB: 1HTN]), contributes to the plasminogen-binding interface [23]—a function enabled by the absence of Ca^2+^-coordinating loop residues that would otherwise constrain this surface for carbohydrate recognition. The conserved Cys152–Cys168 disulfide bond (connecting the loop between β5 and β6 to the C-terminal region adjacent to the α1-helix) stabilizes the CTLD core; disruption of this bond would likely compromise both structural integrity and ligand-binding capacity. The α1-helix (residues 72–82) extends from the CTLD core and may participate in inter-trimer contacts or receptor recognition, though its specific functional role remains to be experimentally defined. Mechanistically, CLEC3B binds plasminogen and its Kringle 4 (K4) domain through two distinct interfaces: the N-terminal lysine-binding site (LBS, residues 23–60) [24,25], which engages plasminogen lysine residues with high affinity; and calcium-independent sites within the CTLD β-sheet surface (centered on residues 27–46, encompassing β2–β4) [17,22]. The bipartite binding mode—combining the N-terminal LBS with the C-terminal CTLD interface—likely enhances binding avidity and specificity, contributing to CLEC3B’s potentiation of tPA-mediated plasminogen activation [26,27]. Through this binding, CLEC3B modulates the activation of plasminogen, thereby participating in the regulation of the fibrinolytic system and the coagulation cascade [20].

Notably, while the CLEC3B CTLD retains the lectin fold, direct experimental evidence for glycan binding remains limited [28]. To date, characterized CLEC3B ligands are predominantly proteinaceous (plasminogen, tPA, HGF, fibronectin, HMGB1). A single study reported agglutinating activity in fish CLEC3B with a predicted galactose-binding site [23]. However, it remains unresolved whether mammalian CLEC3B retains carbohydrate-recognition capacity and whether such activity is physiologically relevant. These questions warrant systematic investigation.

C-type lectins are a class of animal lectins characterized by their Ca^2+^-dependent CRDs, which mediate specific interactions with cell surface glycoconjugates (e.g., glycoproteins, polysaccharides). As key pattern recognition molecules, they can precisely identify various membrane-displayed biomolecules through their conserved CRD modules. CLEC3B, a member of this family, has been identified in humans and mice and is localized in the cellular plasma, the ECM, and exosomes [21]. In humans, the CLEC3B gene was isolated by scientists in 1992 and is located on chromosome 3. CLEC3B, which is encoded by the CLEC3B gene, plays functional roles in a variety of tissues and cells. The CLEC3B gene is also present in mice and is expressed in multiple tissues of mice. Beyond mammals, the CLEC3B gene was also identified in other species. Using transcriptome and BLAST screening, the researchers identified a novel CLEC3B gene in golden pompano [8]. The encoded protein shares similar structural features with its mammalian orthologs, including a conserved CRD. This CTLD comprises two α-helices, six β-strands, and four loops, which together form two Ca^2+^-binding sites and one predicted galactose-binding site. Notably, a putative Ca^2+^-binding site is present in fish CLEC3B that can mediate glycan recognition, whereas mammalian CLEC3B lacks this motif. Whether this represents a bona fide CRD function or an atypical metal coordination remains to be determined. It is normally expressed in a variety of tissues, is significantly upregulated upon challenge by pathogenic microorganisms, and is considered both a component of the host innate immune response and an inducible acute immune factor [8]. Furthermore, protein sequence alignment reveals that the CLEC3B protein shares as high as 90.40% homology across *Homo sapiens*, *Mus musculus*, *Bos taurus*, *Macaca mulatta*, and *Sus scrofa* (Figure 1C). The high homology of the CLEC3B protein across species suggests its critical role in biological processes. Therefore, CLEC3B may be an important gene that plays functional roles in different organisms.

## 3. Molecular Function of CLEC3B

**Figure 2:**
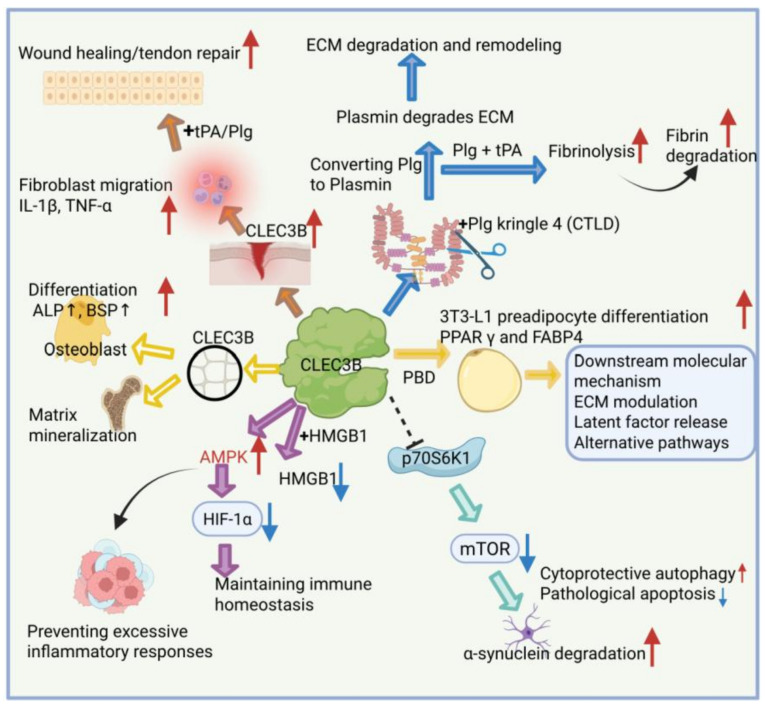
Multifunctional roles of CLEC3B in physiological processes. Tissue repair and regeneration: CLEC3B is upregulated following wound occurrence, promoting fibroblast migration and inflammatory cell infiltration (IL-1β, TNF-α) and synergizing with the tPA/Plg system to facilitate wound healing/tendon repair. Fibrinolytic system regulation: CLEC3B binds to the kringle 4 domain of plasminogen through its C-type lectin domain (CTLD), while also binding to tPA, co-localizing these two molecules to enhance fibrinolysis and promote fibrin degradation. Red upward arrows indicate upregulation or promotion; blue downward arrows indicate downregulation or inhibition. Plasmin degrades ECM components, thereby promoting ECM degradation and remodeling. Bone mineralization: CLEC3B is localized to newly formed trabecular bone, promoting osteoblast differentiation and matrix mineralization, with upregulated expression of alkaline phosphatase (ALP) and bone sialoprotein (BSP). Adipocyte differentiation: CLEC3B promotes differentiation of 3T3-L1 preadipocytes via its C-terminal domain (residues 71–199, encompassing the plasminogen-binding region), upregulating PPARγ and FABP4. This adipogenic activity is structurally dependent on this domain but mechanistically independent of plasminogen activation; downstream mechanisms (ECM modulation, latent factor release, or alternative pathways) remain speculative. Immunomodulation: By binding to HMGB1 to inhibit its release, CLEC3B activates AMPK, which subsequently inhibits HIF-1α activity to maintain immune homeostasis and prevent excessive inflammatory responses. Neuroprotection: Following neuronal internalization, CLEC3B binds to and inhibits p70S6K1, attenuating hyperactive mTOR signaling. This process enhances cytoprotective autophagy, suppresses pathological apoptosis, and promotes α-synuclein degradation. This mechanism has been validated in MPP^+^-treated MN9D cells; however, its in vivo effects and relevance to human disease remain to be established. The exact molecular mechanism underlying the CLEC3B–p70S6K1 interaction is undefined and warrants further investigation. https://BioRender.com/j31wznt (accessed on 21 June 2026).

Current reports on CLEC3B remain incomplete. Existing preclinical studies have shown that CLEC3B appears to play a role in tissue repair and regeneration, regulation of the fibrinolytic system, bone mineralization, adipocyte differentiation, immune regulation, and neuroprotection. These related physiological processes will be discussed in detail in the following sections (Figure 2).

### 3.1. Tissue Repair and Regeneration

Cutaneous wound repair is a complex process involving the formation of granulation tissue, re-epithelialization, and tissue remodeling. These processes are mediated by a large number of growth factors, cytokines, and ECM components. The process of skin wound healing includes the formation of a fibrin- and fibronectin-rich matrix in the wound area, infiltration of neutrophils and macrophages, proliferation of epidermal keratinocytes at the wound margin, formation of granulation tissue, and re-epithelialization [30]. Studies using CLEC3B-knockout mice revealed an important role of CLEC3B in cutaneous wound repair. In the experiments, the back skins of both wild-type mice and CLEC3B knockout mice were treated with a single full-thickness incision (Figure 2). The results demonstrated complete wound healing in wild-type mice by postoperative day 14. Furthermore, CLEC3B expression was found to increase on days 1, 3, and 5 during wound healing in wild-type mice. In contrast, CLEC3B knockout mice exhibited significantly impaired wound healing and failed to achieve complete closure within the same timeframe. The wounds in CLEC3B knockout mice were not healed and not covered with epidermis. These phenotypic differences correlated with CLEC3B expression patterns during healing [31]. At the histological level, the back skin incision of CLEC3B-null mice showed significantly reduced scar tissue and delayed progression of granulation to re-epithelialization compared with wild-type mice. These results suggest that CLEC3B may play a role in the early stages of the wound-healing process [31].

Extending these findings to tendon repair, studies have shown the role of CLEC3B in regulating the formation of tendon tissue after tendon injury [31]. CLEC3B in tendon healing mainly involves the regulation of the inflammatory response and the expression of ECM-related proteins [32]. During the inflammatory phase (within 7 days) after tendon injury, the infiltration of inflammatory cells and the expression of inflammatory cytokines such as IL-1β, IL-6, and TNF-α were significantly reduced in CLEC3B knockout mice compared with wild-type mice [32]. In wild-type mice, CLEC3B expression increased at 1 day after tendon injury, peaked at 3 days, and almost disappeared after 7 days. This suggests that CLEC3B may be involved in initiating and regulating the inflammatory response, which is essential for tendon healing. It can attract immune cells to remove damaged tissue and pathogens, thereby creating the conditions for the subsequent healing process. Experimental evidence indicates that CLEC3B is required for the normal inflammatory phase of tendon repair. In CLEC3B-knockout mice, the early infiltration of inflammatory cells (e.g., macrophages) and the upregulation of key pro-inflammatory cytokines, including IL-1β, IL-6, and TNF-α, are significantly attenuated after injury [32]. This suggests that CLEC3B facilitates the initiation of the inflammatory response, a crucial step for clearing debris and launching subsequent repair processes. The precise signaling pathways through which CLEC3B mediates this immunomodulatory effect remain to be fully elucidated. In CLEC3B knockout mice, tendon healing and maturation are significantly delayed, but they ultimately achieve complete healing. This suggests that CLEC3B also plays a role in the long-term process of tendon healing and that it may help to maintain the continuous advancement of the healing process, ensuring that the tendon tissue can return to a near-normal structural and functional state as soon as possible. After the inflammatory phase, in the later stages of healing, CLEC3B may be involved in regulating cell proliferation and differentiation, as well as in the further remodeling of the ECM through a variety of mechanisms. After the inflammatory phase, CLEC3B facilitates tissue remodeling and maturation through defined mechanisms central to its function as a matricellular cofactor that enhances tPA-mediated plasminogen activation [9]. The principal mechanism involves the potent, localized augmentation of plasmin generation via CLEC3B-mediated co-localization of tissue plasminogen activator and plasminogen. The resulting plasmin executes a multifaceted program essential for repair: it directly cleaves provisional matrix components such as fibrin and remodels structural proteins including fibronectin; it liberates and activates latent growth factors embedded within the extracellular matrix, most notably hepatocyte growth factor; and, through these proteolytic activities, it dynamically reshapes the biochemical and mechanical microenvironment to enable cell migration, proliferation, and differentiation [9]. This orchestrated proteolytic cascade is further fine-tuned by CLEC3B’s ability to modulate signaling pathways such as AMPK, which integrates metabolic and stress responses to ensure the coordinated progression and resolution of the healing process. Through these mechanisms, CLEC3B helps the tendon to gradually restore its mechanical properties and normal function [33]. Collectively, these findings from CLEC3B-knockout mice establish CLEC3B as a putative multi-phase regulator in tendon repair, orchestrating initial inflammatory responses through cytokine modulation, followed by extracellular matrix remodeling during later healing stages, ultimately ensuring functional tissue restoration.

The delayed wound closure in CLEC3B-null mice may reflect direct effects on wound-healing signaling pathways, indirect effects via altered fibrinolysis, or a combination of both; the relative contribution of each mechanism remains experimentally unresolved. Direct effects could involve CLEC3B deficiency-mediated dysregulation of early inflammatory signaling and granulation tissue formation, as supported by the temporal correlation between peak CLEC3B expression (days 1–5) and the initial inflammatory phase [34]. Indirect effects may arise from impaired fibrinolytic activity: CLEC3B deficiency reduces localized plasmin generation by attenuating tPA-mediated plasminogen activation, potentially leading to persistent fibrin deposition and delayed provisional matrix remodeling [35,36]. These mechanisms may not be mutually exclusive—CLEC3B could function as a matricellular hub that potentially integrates proteolytic regulation with cytokine-mediated signaling [34,37]—which awaits further investigation.

### 3.2. Regulation of the Fibrinolytic System

A principal molecular function of CLEC3B is its role as a cofactor in the plasminogen activation system, which is central to ECM degradation and tissue remodeling [23]. CLEC3B has been shown to bind with high affinity to both tissue plasminogen activator (tPA) and the kringle 4 domain of plasminogen (Plg) [23,24]. This interaction potentiates fibrinolysis through a dual mechanism. First, CLEC3B stabilizes the active conformation of bound tPA, protecting it from rapid inhibition or clearance, thereby extending its proteolytic window [6,23]. Second, and perhaps more critically, by simultaneously anchoring tPA and concentrating Plg within the ECM, CLEC3B dramatically enhances their co-localization. This spatial organization increases the local catalytic efficiency of tPA, accelerating the conversion of Plg to active plasmin by approximately an order of magnitude (Figure 2) [6,25]. The kinetic parameters of this CLEC3B-facilitated activation are comparable to those induced by fibrin, the classic physiological cofactor for tPA, suggesting potential physiological relevance of CLEC3B in localized proteolytic events in vitro; however, direct in vivo kinetic evidence is lacking [6].

The functional consequences of this mechanism extend beyond fibrinolysis. Plasmin is a broad-spectrum protease that cleaves ECM components and activates latent growth factors. Therefore, by amplifying plasmin generation, CLEC3B directly facilitates ECM remodeling and creates a permissive environment for cell migration-processes integral to the tissue repair cascades described above. Furthermore, CLEC3B also binds hepatocyte growth factor (HGF). Given that both HGF and tPA can be co-localized in tissues and that tPA is known to activate single-chain HGF, it is plausible that CLEC3B may further coordinate tissue repair by regulating HGF bioavailability, although this hypothesis requires direct experimental validation. Taken together, these interactions position CLEC3B as an interacting hub that converges proteolytic activity and growth factor signaling to orchestrate the spatial and temporal coordination of tissue repair, extending its role from a simple fibrinolytic cofactor to a multifunctional regulator of the regenerative microenvironment.

### 3.3. Role of CLEC3B in the Skeletal System

CLEC3B is a key regulator in skeletal biology, with distinct yet critical functions in bone mineralization and spinal integrity.

#### 3.3.1. Promotion of Osteogenesis and Bone Mineralization

CLEC3B is intimately associated with the mineralization process during bone formation. In newborn mice, it is found to be localized to newly formed woven bone, but not to cartilage or muscle, indicating a specific role in ossification. This spatiotemporal link is recapitulated in vitro [5]. During osteoblast differentiation in mineralization cultures, CLEC3B mRNA expression initiates concurrently with late osteogenic markers and increases alongside mineral deposition. Functional studies confirm its pro-osteogenic role. For instance, tumors formed in nude mice by PC12 cells engineered to overexpress recombinant CLEC3B contained approximately fivefold more bone material than controls, demonstrating a direct positive effect on ectopic bone formation [5]. Mechanistically, knockdown of CLEC3B in osteogenic precursor cells significantly reduces alkaline phosphatase activity, mineralization capacity, and the expression of key regulators like bone sialoprotein (BSP) in these cells (Figure 2). Collectively, these data establish CLEC3B as a bona fide osteogenic factor that promotes osteoblast differentiation and matrix mineralization.

#### 3.3.2. Maintenance of Spinal Architecture and Disc Homeostasis

Beyond its role in bone formation, CLEC3B is essential for maintaining the structural integrity of the spine [38]. Mice with targeted deletion of the CLEC3B gene develop progressive kyphosis (exaggerated thoracic curvature) from 3 to 6 months of age, with a significantly increased thoracic spinal angle compared to wild-type littermates. This deformity is accompanied by aberrant vertebral morphology, such as wedge-shaped bodies [38].

Histological analysis reveals the cellular basis of this phenotype. The spines of CLEC3B-deficient mice exhibit profound abnormalities in the growth plates and intervertebral discs [38]. Growth plates show structural disorganization, asymmetric activity, and abnormal cell morphology, and discs develop asymmetrically, with loose posterior structures and anterior compression in CLEC3B-deficient mice. These defects suggest that CLEC3B is crucial for the coordinated remodeling and maintenance of both the growth plate (responsible for vertebral bone growth) and the disc (responsible for flexibility and cushioning) [38]. The CLEC3B-knockout mouse thus serves as a model for understanding spine stability disorders, sharing features with human conditions like Scheuermann’s kyphosis, a developmental disorder of the spine.

### 3.4. Role of CLEC3B in Adipocyte Differentiation

CLEC3B functions as a factor promoting adipogenesis, an activity mapped to its plasminogen-binding domain (PBD). Proteomic analyses first identified CLEC3B as an adipogenic factor in serum [39]. Subsequent functional studies confirmed that culture medium depleted of CLEC3B impairs the differentiation of 3T3-L1 preadipocytes, a defect rescued by supplementing with recombinant mouse CLEC3B (rmCLEC3B) [40]. CLEC3B comprises three structural domains, namely Domain 1, Domain 2, and Domain 3, each possessing unique structural and functional characteristics that collectively mediate various physiological roles of CLEC3B in living organisms. Domain 1 is typically situated at the N-terminal region of CLEC3B, and its core structural feature is a coiled-coil domain formed by α-helices. This structure is commonly found in many proteins, primarily functioning to mediate protein–protein interactions, forming homo- or heteromultimers. Domain 2 is generally located after Domain 1 and is also primarily composed of α-helical structures. The specific functions of this domain can be diverse, but it is widely believed to contribute to maintaining the overall conformational stability and flexibility of the protein. Domain 3 is located at the C-terminal region of CLEC3B and contains its core CTLD. This domain is crucial for the biological function of CLEC3B. Functional mapping using domain-deletion constructs revealed that the pro-adipogenic activity resided entirely within the C-terminal region. Only recombinant proteins encompassing the plasminogen-binding domain (PBD, located in Domain 3)—whether in the context of the full-length protein recombinant mouse CLEC3B (rmCLEC3B123), a truncated form retaining the helical and C-terminal domains (rmCLEC3B23), or the isolated C-terminal domain itself (rmCLEC3B3)—could promote differentiation. A construct lacking this domain (rmCLEC3B12) was ineffective, unequivocally identifying the PBD as the critical functional domain [40]. This pro-adipogenic role is further supported by the upregulation of key differentiation markers (PPARγ, FABP4, and CD36). This upregulation was observed following treatment with PBD-containing CLEC3B.

Notably, this function appears independent of the canonical plasminogen activation cascade, as direct addition of Plg, tPA, or plasmin did not mimic CLEC3B’s effect on promoting adipogenesis [40]. The precise mechanism remains unknown. One plausible hypothesis is that CLEC3B, through its PBD, may influence the ECM microenvironment in a plasmin-independent manner. Since ECM remodeling is a prerequisite for adipocyte expansion, CLEC3B could hypothetically facilitate differentiation by modulating cell-ECM interactions or the availability of latent adipogenic factors within the matrix. This model aligns with the known role of other matrix-associated proteins in adipogenesis [41], yet further verification is still required.

### 3.5. Role of CLEC3B in the Inflammatory Response and in the Immune Regulation

**Figure 3:**
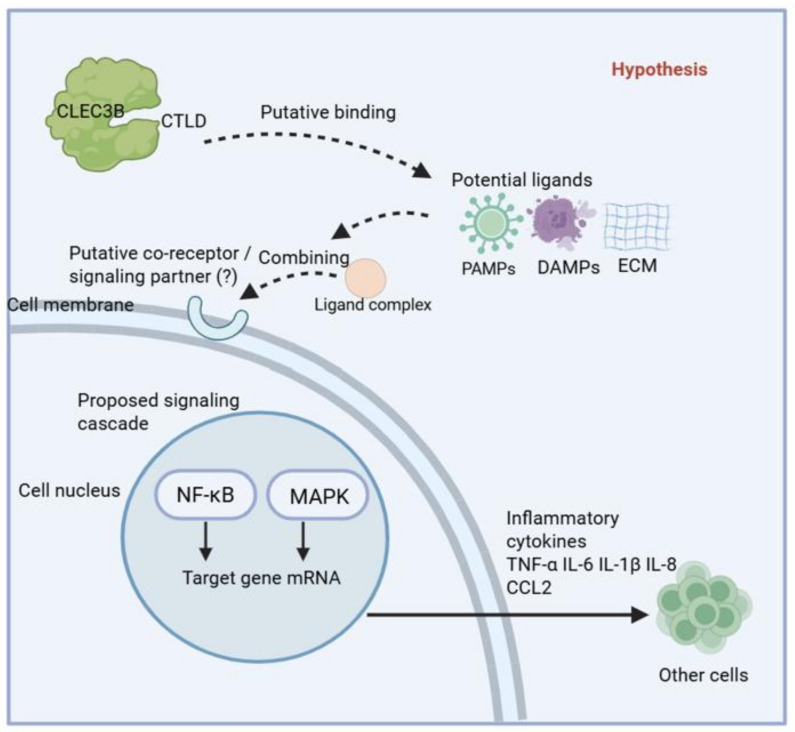
Proposed working model of CLEC3B-mediated inflammatory signaling. This figure presents a working hypothesis based on current evidence. CLEC3B contains a C-type lectin-like domain (CTLD) and is hypothesized to recognize pathogen-associated molecular patterns (PAMPs), damage-associated molecular patterns (DAMPs), and extracellular matrix (ECM) components. However, direct evidence for CLEC3B functioning as a pattern-recognition receptor remains limited. The downstream signaling pathway involving an unidentified receptor and subsequent NF-κB/MAPK activation is speculative and requires experimental validation. The induction of inflammatory cytokines (TNF-α, IL-6, IL-1β, IL-8, CCL2) represents a predicted outcome of this proposed signaling axis. Solid arrows indicate established protein domains/secretory processes; dashed arrows indicate hypothetical interactions requiring further investigation. https://BioRender.com/u201rkx (accessed on 21 June 2026).

Beyond its role in ECM organization, CLEC3B directly modulates innate immune responses. It functions as a binding protein for High Mobility Group Box 1 (HMGB1), a key damage-associated molecular pattern (DAMP). This interaction has functional consequences: purified CLEC3B protein can inhibit lipopolysaccharide (LPS)-induced HMGB1 release from macrophages in a dose-dependent manner [41]. The clinical relevance of this regulatory axis is underscored by the observation that circulating CLEC3B levels plummet in patients with severe sepsis, suggesting its consumption or dysregulation contributes to the hyperinflammatory state. Thus, CLEC3B may act as an endogenous brake on excessive DAMP-driven inflammation during infection (Figure 3).

CLEC3B dysregulation is also a feature of sterile inflammation and metabolic diseases. In severe COVID-19, proteomic analyses identified CLEC3B as a significantly downregulated protein, linking it to viral pathogenesis and systemic inflammation [42]. This deficiency may be intertwined with vascular dysfunction, a common complication in severe cases. Notably, CLEC3B serum levels often decrease concurrently with paraoxonase 1 (PON1), an antioxidant enzyme associated with HDL [43]. PON1 exerts its cardioprotective effect by hydrolyzing lipid peroxides to protect low-density lipoprotein (LDL) and cell membranes from oxidative damage. The decrease in PON1 activity is an important biomarker of cardiovascular diseases (such as atherosclerosis). PON1-mediated hydrolysis of oxidized lipids is cardioprotective, and its decline is a biomarker for cardiovascular risk. The co-reduction in CLEC3B and PON1 activities in conditions such as atherosclerosis suggests that their pathways may converge in mitigating oxidative stress and inflammation [44]. For instance, PON1 is known to temper macrophage hyperactivation, and CLEC3B deficiency might exacerbate a similar pro-inflammatory milieu [45].

An integrative function of CLEC3B involves modulation of the AMPK-HIF-1α signaling axis, which may coordinate angiogenesis and innate immunity [2,46]. Under normal conditions, CLEC3B has been reported to enhance AMP-activated protein kinase (AMPK) activity by promoting its phosphorylation at Thr172 [47]. This activation is associated with targeting hypoxia-inducible factor-1α (HIF-1α) for proteasomal degradation, thereby restraining vascular endothelial growth factor (VEGF) synthesis and angiogenesis [48]. Loss or downregulation of CLEC3B has been linked to disruption of this homeostatic circuit, leading to attenuated AMPK activity and subsequent HIF-1α stabilization [49]. Consequently, VEGF expression may be derepressed, potentially driving pathological neovascularization—a hallmark of cancer and other diseases. Additionally, HIF-1α stabilization has been proposed to skew macrophage polarization towards a pro-inflammatory, M1-like phenotype while impairing dendritic cell maturation, alterations that may facilitate immune evasion [50]. Therefore, CLEC3B deficiency may contribute to a pathological loop involving aberrant angiogenesis and an immunosuppressive milieu, a hypothesis primarily derived from correlative and cell-based studies suggesting that CLEC3B might be a candidate regulator of the AMPK–HIF-1α axis involved in suppressing carcinogenesis by affecting angiogenesis and innate immunity.

### 3.6. Neuroprotective Role of CLEC3B: Inhibition of Apoptosis and Promotion of Autophagy

Evidence implicates CLEC3B as a neuroprotective factor, particularly in the context of Parkinson’s disease (PD). Initial clinical observations revealed a significant decrease in CLEC3B levels in the cerebrospinal fluid (CSF) of PD patients compared to healthy controls, with partial recovery following therapeutic deep brain stimulation [51,52]. This clinical correlation is reinforced by in vivo evidence: CLEC3B-knockout mice develop motor deficits and pathological features reminiscent of PD, including dopaminergic neuron loss in the substantia nigra and enhanced neuronal apoptosis [11]. This is speculated to result from enhanced apoptosis specifically within the dopaminergic neurons of the substantia nigra pars compacta.

To elucidate the underlying molecular mechanism, researchers focused on the secreted form of CLEC3B. Bioinformatic analyses of protein interaction databases suggested ribosomal protein S6 kinase beta-1 (p70S6K1), a key downstream effector of the mTOR pathway governing cell survival, growth, and autophagy, as a potential intracellular target for CLEC3B (Figure 2). Subsequent experimental validation using the mouse dopaminergic neuronal cell line MN9D confirmed this hypothesis. Exogenous administration of CLEC3B protein alleviated neurotoxicity and reduced oxidative stress induced by the parkinsonian neurotoxin MPP^+^ [53]. Mechanistically, CLEC3B is internalized by MN9D cells and directly interacts with p70S6K1, reducing its phosphorylation at Thr389. In the context of MPP^+^-induced neurotoxicity, p70S6K1/mTOR signaling is hyperactivated, which impairs autophagic flux by suppressing lysosomal biogenesis and autophagosome–lysosome fusion [53,54]. By attenuating this hyperactive p70S6K1/mTOR axis, CLEC3B restores functional autophagic flux, enabling efficient clearance of damaged mitochondria and protein aggregates [55]. This restoration of autophagic homeostasis contributes to neuronal survival by preventing both the accumulation of toxic aggregates (due to insufficient autophagy) and the energy depletion/cell death associated with stalled autophagic flux [55]. This mechanism provides a proposed molecular basis for the neuroprotective phenotype observed in a single mouse model and highlights CLEC3B as a preclinical therapeutic candidate for neurodegenerative disorders.

### 3.7. CLEC3B as a Protein That Orchestrates ECM Dynamics

Beyond its individual ligand-binding activities, CLEC3B functions as a matrix-associated protein. This class of molecules does not primarily provide structural integrity to the ECM but dynamically modulates cell–ECM communication to guide tissue development, repair, and homeostasis [6]. CLEC3B exemplifies this role through its multifaceted interactions, integrating several key regulatory axes: structural cross-linking and assembly, enzymatic activity regulation, and signal storage and presentation.

#### 3.7.1. Structural Cross-Linking and Assembly

CLEC3B directly binds fibronectin, a major ECM glycoprotein, and can alter its fibrillar assembly. This interaction allows CLEC3B to modify the physical scaffold, directly affecting the mechanical signals that cells receive from their extracellular environment during adhesion and migration.

#### 3.7.2. Enzymatic Activity Regulation

CLEC3B exhibits high-affinity binding to both Plg and tPA. Through this dual interaction, it effectively recruits and concentrates these molecules at specific anchoring sites, such as the cell surface, extracellular matrix (ECM), and fibrin clots [9]. This spatial aggregation and elevated local concentration significantly enhance the generation of plasmin. By spatially regulating the activation of plasminogen, thereby modulating the activity of plasmin, a broad-spectrum protease, CLEC3B promotes the cleavage of ECM components (such as fibrin and laminin) and participates in the activation of latent growth factors (such as HGF and TGF-β), thereby driving local ECM degradation and turnover, ultimately facilitating ECM degradation and angiogenesis, and potentially playing a role in pathological processes such as tumor invasion and metastasis (Figure 2) [9].

#### 3.7.3. Signal Storage and Presentation

CLEC3B has been shown to directly bind hepatocyte growth factor (HGF) with micromolar affinity and to accumulate tissue-type plasminogen activator (tPA) in an active conformation, enhancing its catalytic activity. In addition to these direct protein–protein interactions, CLEC3B binds calcium ions at sites that allosterically regulate its conformation and ligand-binding specificity [56]. Although CLEC3B does not mediate canonical Ca^2+^-dependent carbohydrate recognition, its calcium-binding capacity contributes to heparin interactions that may facilitate co-localization with heparin-binding growth factors in the ECM [19,57]. Heparan sulfate proteoglycans (HSPGs) in the ECM are known to bind and stabilize a broad spectrum of heparin-binding growth factors, including VEGF, FGFs, and BMPs [5,23]. Through its heparin-binding domain, CLEC3B may co-localize with these HSPG-bound growth factors within the ECM, potentially contributing to their localized concentration and presentation to cell surface receptors [19]. These create localized reservoirs of bioactive molecules, making them available for presentation to cell-surface receptors in a spatially and temporally controlled manner, which is crucial for processes such as osteogenesis and angiogenesis [23]. Whether CLEC3B-mediated co-localization with various factors occurs in the context of ECM-based presentation in vivo remains to be directly demonstrated.

Collectively, through the three complementary axes above, CLEC3B dynamically reprograms the extracellular matrix. This reprogramming directly alters the biomechanical properties (e.g., stiffness, topography) and the biochemical ligand landscape of the cellular microenvironment. These changes are, in turn, transduced into intracellular signals that govern fundamental cell behaviors such as differentiation, proliferation, and survival, thereby manifesting CLEC3B’s pleiotropic effects in multiple processes, including adipogenesis, osteogenesis and tumor progression.

CLEC3B functions as an interacting hub within the ECM network, concurrently engaging structural proteins (fibronectin), proteolytic enzymes (tPA/Plg), and signaling cofactors (heparin/Ca^2+^). This multi-ligand binding capacity provides a mechanistic basis for its pleiotropic effects, though the relative contribution of each interaction to specific pathological outcomes remains to be quantitatively determined.

## 4. CLEC3B in Diseases

CLEC3B exhibits distinct disease-specific associations. In cardiovascular diseases, CLEC3B is implicated in tissue remodeling and may mark disease progression: it is upregulated in cardiac fibrosis and post-infarction remodeling, promoting extracellular matrix deposition; circulating levels correlate with adverse cardiac remodeling severity. In autoimmune diseases, CLEC3B influences both disease susceptibility and tissue destruction. Genetic variants and dysregulated expression are linked to an increased risk of rheumatoid arthritis and SLE, whereas local CLEC3B signaling contributes to the exacerbation of synovial inflammation and joint architectural damage. In cancer, CLEC3B serves as a prognostic biomarker and mediates tumor microenvironment remodeling: stromal expression predicts poor survival and metastasis, while circulating forms enable non-invasive prognostic assessment; mechanistically, it promotes desmoplastic reactions supporting tumor progression.

### 4.1. The Role of CLEC3B in Osteoarthritis

CLEC3B plays a pivotal role in maintaining skeletal homeostasis, with its dysregulation contributing to both degenerative joint diseases and impaired bone formation.

**Figure 4:**
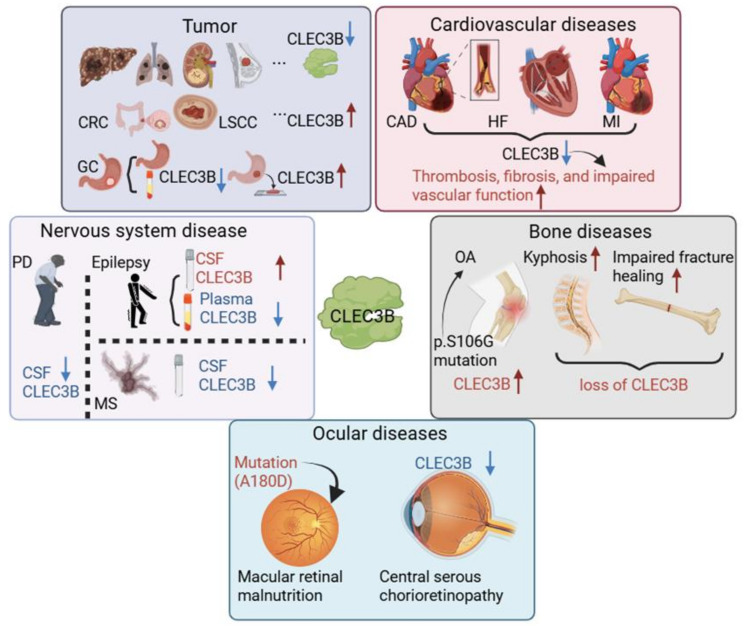
Expression changes and functional roles of CLEC3B in multiple diseases. Tumor diseases: CLEC3B is downregulated in most solid tumors (HCC, lung, renal, breast cancer) as a tumor suppressor via EMT, AMPK–VEGF, and Wnt/β-catenin pathways. In CRC, LSCC, and intratumoral GC, CLEC3B is upregulated and promotes tumor progression via ECM degradation and angiogenesis. Red upward arrows indicate upregulation or promotion; blue downward arrows indicate downregulation or inhibition. Cardiovascular diseases: CLEC3B is downregulated in CAD, HF, and MI. Its fibrinolytic role is established; TGF-β/Smad and PI3K/Akt/eNOS mechanisms are proposed. Nervous system disease: Reduced CSF CLEC3B in PD is established; neuroprotection via p70S6K1 inhibition is demonstrated. Epilepsy shows dissociated CSF/serum patterns; MS shows reduced CSF levels. Bone diseases: CLEC3B is elevated in OA (p.S106G mutation associated with susceptibility); bone repair via osteoblast differentiation is demonstrated; a kyphosis phenotype upon deletion is established. Ocular diseases: The A180D mutation causes established macular retinopathy; CSCR downregulation is based on preliminary data. https://BioRender.com/vcavkj7 (accessed on 21 June 2026).

Osteoarthritis (OA) is a major source of pain, disability, and socioeconomic cost worldwide. The epidemiology of the disorder is complex and multifactorial, with genetic, biological, and biomechanical components. Etiological factors are also joint-specific. Joint replacement is an effective treatment for symptomatic end-stage disease, although functional outcomes can be poor and the lifespan of prostheses is limited. Consequently, the focus is shifting to disease prevention and the treatment of early OA. CLEC3B is implicated in the pathogenesis of OA, a prevalent degenerative joint disorder. A key genetic association study identified the CLEC3B missense variant p.S106G as being linked to susceptibility for knee OA [58]. This genetic link is supported by expression data: transcriptomic analyses consistently show that CLEC3B mRNA levels are significantly elevated in the articular cartilage of OA patients compared to healthy controls (Figure 4). The functional significance of this upregulation remains an active area of investigation. Given the established role of CLEC3B in plasminogen activation and ECM remodeling (see Section 3), its overexpression in OA cartilage may contribute to aberrant matrix turnover, abnormal chondrocyte signaling, or the low-grade inflammatory milieu characteristic of the disease [59].

Beyond its potential role in cartilage-related diseases, CLEC3B is a direct positive regulator of osteogenesis. During the differentiation of human mesenchymal stem cells (hMSCs) into osteoblasts, CLEC3B expression is markedly upregulated [60]. Functional loss-of-function experiments confirm its necessity: knockdown of CLEC3B in osteoprogenitor cells significantly reduces alkaline phosphatase activity, diminishes the expression of late mineralization markers like BSP, and severely impairs matrix mineralization capacity [60]. These findings align with earlier in vivo observations that CLEC3B localizes to sites of active bone formation and promotes ectopic mineralization. Collectively, the evidence positions CLEC3B as a critical anabolic factor in bone, necessary for osteoblast function and proper mineral deposition. Its deficiency or dysfunction could therefore underlie or exacerbate conditions characterized by low bone mass or impaired fracture healing (Figure 4).

The dual involvement of CLEC3B in cartilage integrity and bone formation underscores its broad importance in the musculoskeletal system. Its genetic association with OA and its essential role in osteoblast biology make it a compelling molecule for understanding the shared and distinct pathologies of joint degeneration and skeletal fragility.

### 4.2. The Role of CLEC3B in Skeletal Muscle Atrophy

CLEC3B exhibits characteristics as a potential molecular marker in skeletal muscle atrophy, with its expression changes correlating with disease progression and demonstrating differential expression patterns across various myopathic conditions [53,61]. In a mouse model of spinal muscular atrophy (SMA), plasma or serum levels of CLEC3B have been reported to be significantly upregulated, and this elevation appears to be associated with disease severity and progression [62,63]. However, plasma CLEC3B expression shows no significant correlation with the clinical phenotype of SMA, which may limit its utility for individual patient monitoring [64]. In contrast, transcriptomic and proteomic analyses of skeletal muscle atrophy models in mice have identified CLEC3B as a differentially expressed molecule, with reduced expression in atrophic muscle tissue [64,65]. These differential expression patterns suggest that CLEC3B may function as a context-dependent biomarker, with its directionality varying across disease types and biological compartments. Whether CLEC3B can serve as a reliable biomarker for muscle atrophy requires further validation in defined clinical contexts.

### 4.3. Involvement of CLEC3B in Nervous System Diseases

CLEC3B can be detected in the CSF. CSF is primarily produced by the choroid plexus epithelium, with minor contributions from trans-barrier filtration and possible paracrine secretion by glial/neuronal cells [66,67]. Although the composition of cerebrospinal fluid is strictly regulated under physiological conditions, its molecular profile can be significantly altered in neurological diseases. In epilepsy, CLEC3B concentration increases in CSF but decreases in serum [68], demonstrating disease-specific alteration patterns (Figure 4). CLEC3B shows disease-specific patterns of changes in neurological diseases: elevated in epileptic CSF through an activity-dependent secretion mechanism; decreased in chronic diseases such as Multiple Sclerosis (MS), which is related to blood–brain barrier (BBB) damage (Figure 4). This difference suggests that it has unique pathophysiological effects in the different nervous system disease spectrum [69]. Proteomic analysis has consistently identified CLEC3B as a protein downregulated in the CSF of PD patients (Figure 4) [51]. Intriguingly, its CSF level can be transiently increased by therapeutic deep brain stimulation and decrease upon treatment withdrawal, suggesting a dynamic association with neuronal activity or therapeutic modulation [70]. In vivo studies showed that CLEC3B knockout in mice led to the development of PD and enhanced neuronal apoptosis [11,46]. Conversely, genetic studies have identified a potentially protective role for a CLEC3B variant. The missense variant c.316G>A (p.S106G, rs13963) has been associated with extended lifespan and maintained neurological health in aging populations, suggesting it may confer a protective advantage [71]. In the PD brain, the main component of Lewy bodies is α-synuclein; its aggregation plays a key role in neuronal degeneration, and its cell-to-cell transmission drives disease progression. Recent studies have shown that exogenous CLEC3B, when introduced into model cell lines, can effectively reduce the accumulation of α-synuclein aggregates, which are characteristic of synucleinopathies such as PD. Additionally, CLEC3B can inhibit the cell-to-cell transmission of α-synuclein, thereby potentially slowing the progression of these neurodegenerative diseases [11]. This study showed that CLEC3B promoted the degradation of α-syn by activating the plasminogen activation system, revealing the interaction between CLEC3B, plasmin, and α-syn, which also explained the molecular mechanism of CLEC3B in inhibiting the disease progression of PD.

### 4.4. Role of CLEC3B in Tumor-Related Diseases

Cancer arises from aberrant gene regulation, including the upregulation of oncogenes and the downregulation of cancer suppressor genes. CLEC3B demonstrates context-dependent roles in tumor progression. Functionally, CLEC3B displays a striking duality in oncology. On the one hand, the tumor-suppressive function of CLEC3B has been demonstrated in various cancers, such as hepatocellular carcinoma (HCC) [2]. On the other hand, CLEC3B may function as a tumor promoter in the oncogenesis of some tumors, such as cancer-related lymphedema (CAL) and colorectal cancer [2,4,72]. This suggests that CLEC3B may exert opposite functions depending on the tumor type or tumor microenvironment [73]. 

CLEC3B has been proposed to play a crucial role as a tumor suppressor gene in the occurrence and development of HCC based on in vitro and animal studies; however, its classification as a bona fide tumor suppressor gene awaits genetic validation in human tumors [2,74]. Its functional inactivation or downregulation drives the malignant progression, immune escape, and treatment resistance of HCC through multiple molecular mechanisms. One mechanism involves exosome-mediated transfer of CLEC3B: HCC-derived extracellular vesicles with low CLEC3B levels are internalized by tumor cells or endothelial cells, leading to reduced AMPK phosphorylation and consequent VEGF upregulation [2]. This exemplifies the functional significance of exosomal CLEC3B as a cell-to-cell signaling mediator, distinct from the broad ECM reservoir of secreted CLEC3B. The uptake of HCC-derived extracellular vesicles with low levels of CLEC3B by tumor cells or endothelial cells inhibits the phosphorylation of intracellular AMPK, thereby upregulating the expression of VEGF [2]. On the one hand, this directly promotes EMT in HCC cells, significantly enhancing their migration and invasion abilities; on the other hand, it stimulates tumor angiogenesis, providing essential support for tumor growth and metastasis, and ultimately accelerating the malignant progression and distant metastasis of HCC [75].

In addition, loss of CLEC3B indirectly affects the pathological process of HCC by reshaping the tumor immune microenvironment (TME) and regulating matrix components [72]. In terms of immune regulation, low CLEC3B expression is associated with reduced infiltration and functional inhibition of anti-tumor immune cells, such as CD8+ T cells and NK cells [76]. At the same time, it may promote the expression of immune checkpoint molecules such as PD-L1 by activating the STAT3 pathway and increasing the recruitment of myeloid suppressor cells (MDSCs), jointly creating an inhibitory microenvironment conducive to tumor immune escape. In terms of TME matrix regulation, the expression of CLEC3B is inhibited by factors secreted by cancer-associated fibroblasts (CAFs), such as TGF-β1 [77,78]. Its low expression further activates survival-promoting pathways such as PI3K/Akt, leading to HCC cells developing drug resistance to targeted drugs such as sorafenib and lenvatinib. Meanwhile, CLEC3B expression is positively correlated with matrix score, and its absence may lead to a loose extracellular matrix structure, providing convenience for tumor cell invasion. In summary, as a multifunctional molecular node, the downregulation of CLEC3B expression systematically promotes the pathological development of HCC from multiple aspects, such as promoting the malignant phenotype of tumor cells, helping tumors escape immune surveillance, and inducing treatment resistance [79,80].

*CLEC3B* appears to play a major role as a tumor suppressor gene in the pathological progression of lung tumors, mainly including lung adenocarcinoma (LUAD) and non-small cell lung cancer (NSCLC) [81]. Its downregulation drives the invasion, metastasis, immune escape, and treatment resistance of lung cancer through two pathways: directly regulating the malignant phenotype of tumor cells and indirectly reshaping the TME [82,83,84].

In lung tumors, from the perspective of tumor cell-autonomous effects, CLEC3B normally inhibits EMT and cell invasion. Downregulation of CLEC3B expression directly leads to the downregulation of the epithelial marker E-cadherin and activates the key EMT transcription factors Snail/Twist, thereby promoting tumor cells to acquire a mesenchymal phenotype, significantly enhancing their migration and invasion capabilities [85]. This process has been confirmed in in vitro experiments and animal models. Meanwhile, the expression of CLEC3B is also regulated by epigenetics, and hypermethylation of its promoter region is an important mechanism underlying its inactivation. Treatment with demethylating drugs can restore its expression and inhibit tumor growth [10,85]. In terms of tumor microenvironment remodeling, low CLEC3B expression promotes the formation of an immunosuppressive microenvironment [85]. On the one hand, it inhibits anti-tumor immunity by activating signaling pathways such as NF-κB/STAT3, increasing the release of pro-inflammatory factors (e.g., TNF-α, IL-6) and the expression of the immune checkpoint molecule PD-L1, while promoting M2 macrophage polarization and the recruitment of MDSCs [86]. On the other hand, downregulation of CLEC3B enhances the detrimental crosstalk between tumor cells and stromal cells, such as CAFs and monocytes, promotes stromal activation and inflammatory responses through signaling pathways such as MIF/TGF-β, and provides support for tumor progression [86]. These changes collectively lead to reduced responsiveness to immune checkpoint inhibitor therapy. In addition, the expression level of CLEC3B is closely associated with therapeutic sensitivity. Its low expression is correlated with increased resistance to chemotherapeutic agents such as gemcitabine and cisplatin, as well as EGFR tyrosine kinase inhibitors including gefitinib [87,88]. In conclusion, as a multifunctional molecular node, the inactivation of CLEC3B systematically promotes the malignant progression of lung tumors at multiple levels, including enhancing tumor cell invasiveness, establishing an immunosuppressive barrier, and inducing therapeutic resistance.

In biliary tract cancers, CLEC3B expression level is universally downregulated. It exerts a definitive tumor-suppressive role in cholangiocarcinoma (CCA), whereas its significance in gallbladder cancer (GBC) lies primarily in its diagnostic value, with its functional role remaining to be elucidated [89,90]. In CCA, downregulation of CLEC3B expression is a critical pathological feature. Functional studies demonstrate that CLEC3B exerts its anticancer effects by inhibiting the classical Wnt/β-catenin signaling pathway: upregulation of CLEC3B expression reduces the phosphorylation level of GSK-3β, promotes β-catenin degradation, and thereby blocks the activation of this pathway [89]. This mechanism directly suppresses the proliferation and clonal formation capacity of CCA cells and induces apoptosis. Additionally, CLEC3B upregulates E-cadherin and downregulates N-cadherin, inhibiting EMT, which significantly reduces the migration and invasion of CCA cells. Notably, as a calcium-binding protein, its anticancer activity can be synergistically enhanced by Ca^2+^, suggesting that local microenvironmental ion concentrations may influence its function [89]. In vivo experiments further confirm that overexpression of CLEC3B significantly inhibits the growth of transplanted tumors in nude mice. In GBC, although experimental functional evidence remains to be supplemented, multi-omics data analysis consistently indicates that CLEC3B is one of the key genes with significantly downregulated expression and has been identified as a potential diagnostic biomarker. Bioinformatics analysis suggests that CLEC3B may participate in the progression of GBC through pathways related to cell adhesion, EMT, and extracellular matrix remodeling (e.g., synergistic interactions with genes such as SLIT3 and COL7A1) [91]. In conclusion, in biliary tract cancers, CLEC3B, as a molecule with universally downregulated expression, has been demonstrated to exert a definitive anticancer effect in CCA by inhibiting the Wnt/β-catenin pathway, while exhibiting significant diagnostic value in GBC. Its specific mechanisms warrant further investigation [89].

CLEC3B primarily functions as a tumor suppressor gene in the pathological progression of pancreatic ductal adenocarcinoma (PDAC) [92]. Its expression is significantly downregulated in tumor tissues and metastatic foci, with low expression associated with poor prognosis in patients. This downregulation may be regulated by epigenetic mechanisms such as DNA methylation. CLEC3B directly inhibits the migration and invasion capacity of pancreatic cancer cells. More importantly, it plays a profound role in reshaping the unique tumor microenvironment of pancreatic cancer [93,94]. On the one hand, the expression of CLEC3B is positively correlated with the infiltration of anti-tumor immune cells (e.g., CD4+ T cells and CD8+ T cells), suggesting its immunomodulatory potential. On the other hand, the expression of CLEC3B in stromal fibroblasts is specifically regulated by estrogen signaling: estrogen synthesized within the tumor can induce high expression of stromal CLEC3B in stromal fibroblasts, thereby shaping a stromal environment with anti-fibrotic and tumor-inhibitory properties [95]. This partially explains the phenomenon that female pancreatic cancer patients often have a better prognosis. Additionally, CLEC3B can be transmitted between cells via exosomes, taken up by vascular endothelial cells, and may inhibit tumor angiogenesis [2,79]. Clinically, decreased plasma CLEC3B levels are associated with an increased risk of PDAC and, when combined with conventional biomarkers such as CA19-9, may serve as a complementary early diagnostic biomarker to improve detection sensitivity and specificity.

CLEC3B primarily functions as a tumor suppressor gene in the pathological process of renal clear cell carcinoma (ccRCC) [96]. Its characteristic expression is significantly downregulated, which is closely associated with the malignant progression and poor prognosis of ccRCC. In-depth studies have revealed that this downregulation is mainly driven by deep copy number deletion (CNDE), with a deletion rate as high as approximately 88%, and is highly correlated with the hallmark event of ccRCC—the von Hippel–Lindau tumor suppressor (VHL) gene deletion—suggesting a potential co-deletion of the two genes [96]. Functionally, CLEC3B can directly inhibit the proliferative capacity of ccRCC cells, and its molecular mechanism involves key regulation of the MAPK signaling pathway: overexpression of CLEC3B significantly suppresses the phosphorylation of p38 and ERK kinases, thereby blocking the signaling of this pro-proliferative pathway [96]. Additionally, the expression pattern of CLEC3B is positively correlated with a series of genes involved in negative regulation of the cell cycle and apoptosis, collectively forming a molecular network that inhibits tumor growth. In summary, CLEC3B is a key tumor suppressor in ccRCC that is inactivated by copy number deletion and inhibits tumor cell proliferation by suppressing the MAPK pathway, serving as an important negative regulatory molecule in the development and progression of ccRCC [96].

In breast cancer (BC), CLEC3B primarily functions as a tumor suppressor gene. Its expression is significantly downregulated in breast cancer tissues, particularly in samples associated with bone metastasis, and its low expression is correlated with poor patient prognosis [97]. Functionally, CLEC3B directly inhibits the proliferation, migration, and invasive capacity of breast cancer cells. The mechanisms include upregulating E-cadherin, downregulating stromal markers, and EMT-related transcription factors, thereby blocking the EMT process. Notably, CLEC3B, which encodes tetranectin, has been identified as playing a key role in immune-related regulation that impacts bone metastasis [98]. It exerts its inhibitory effects by synergizing with SLIT2 to suppress the activation of the P38 MAPK/c-Fos signaling pathway, thereby inhibiting tumor cell colonization and growth in the bone microenvironment. Additionally, the expression of CLEC3B is positively correlated with the infiltration of anti-tumor immune cells (e.g., CD8+ T cells and M1 macrophages), suggesting its role in modulating the tumor immune microenvironment [99].

In cervical cell cancer (CCC), CLEC3B also functions as a tumor suppressor. Its downregulation is closely associated with high methylation in the promoter region, and low expression is an independent risk factor for poor prognosis, correlating with an increased risk of lymph node metastasis [100]. One of the core roles of CLEC3B is to regulate the tumor immune microenvironment: its high expression is positively correlated with increased infiltration of anti-tumor immune cells such as B cells and CD4+ T cells, contributing to the establishment of an immunologically activated environment [101]. Conversely, its loss of expression or mutation exacerbates immunosuppression. CLEC3B has also been identified as a core immune gene associated with tumor mutation burden (TMB), and prognostic models based on it can effectively predict patient survival. At the mechanistic level, as a key component of the exocytosis pathway, functional abnormalities in CLEC3B may affect cytokine secretion and extracellular matrix remodeling, thereby promoting the invasion and metastasis of cervical cancer [101].

Gastric cancer (GC) exemplifies the ECM-dependent functional duality of CLEC3B, as the protein has been reported to exert both tumor-suppressive and tumor-promoting effects depending on the local ECM context. In the systemic compartment (plasma), CLEC3B downregulation serves as a risk biomarker, consistent with its tumor-suppressive role. However, in the local tumor microenvironment, aberrantly high intratumoral CLEC3B—likely derived from both tumor cells and activated stroma—promotes aggressive phenotypes through enhanced plasminogen-dependent ECM degradation and angiogenesis [102]. Compared with normal gastric mucosa, the overall expression of CLEC3B is downregulated in GC tissue and gradually decreases with the progression of precancerous lesions [103,104]. Notably, while CLEC3B is generally downregulated in GC tissues, a subset of tumors with abnormally high CLEC3B expression exhibits a more aggressive phenotype (e.g., deep infiltration, lymph node metastasis, poor prognosis), forming a unique “high-expression paradox” [102]. In terms of molecular mechanism, this pro-cancer effect mainly stems from the fact that its encoded protein (tetranectin), which functions as a cofactor that enhances the plasminogen–plasmin system. This activation in turn promotes extracellular matrix degradation and angiogenesis [105]. In the special scenario of peritoneal metastasis, low expression of CLEC3B is associated with increased risk of metastasis, and its mechanism may involve promoting the activation of cancer-associated fibroblasts (CAFs) and mediating resistance to anoikis (a form of apoptosis induced by cell detachment) [103]. At the plasma level, low levels of CLEC3B are an independent warning signal for increased risk of GC, making it a key negatively correlated member in the multi-protein risk scoring model, demonstrating potential for early screening in large-scale populations [103]. Therefore, the role of CLEC3B in GC is highly dependent on its temporal and spatial context of expression: in the local tumor microenvironment, its aberrantly high expression may drive aggressive phenotypes; in the circulatory system, its low level can be used for risk prediction [102,103].

In metastatic oral cancer, the anticancer effect of CLEC3B is reflected in its role as a metastasis-monitoring biomarker [78]. The levels of CLEC3B in patients’ serum and saliva are significantly reduced during metastasis, and their low expression is associated with shorter survival [106]. Functionally, CLEC3B may inhibit the resistance of tumor cells to apoptosis by downregulating matrix metalloproteinases (such as MMP-2 and MMP-9) and affecting signaling pathways such as PI3K/AKT, thereby hindering their survival and distant colonization in the bloodstream [78]. In oral squamous cell carcinoma (OSCC), CLEC3B tends to play an anticancer role. Its expression is significantly downregulated in tumor tissue, serum, and saliva, and low expression is associated with lymph node metastasis and poor prognosis [107]. The core mechanism involves two aspects: on the one hand, CLEC3B(tetranectin) may sequester plasminogen through its lysine-binding site, limiting its conversion to active plasmin andthereby reducing extracellular matrix degradation and limiting the invasion and metastasis of tumor cells; on the other hand, its expression level is positively correlated with the infiltration of anti-tumor immune cells (such as CD8+ T cells and M1 macrophages), which helps maintain the immune-active microenvironment [107]. Based on this, CLEC3B was included in a multi-gene prognostic model for OSCC patient risk stratification.

In head and neck squamous cell carcinoma (HNSCC), CLEC3B is one of the core downregulated genes, and its expression level has important subtyping value [108]. Based on six genetic features, including CLEC3B, HNSCC can be classified into subtypes with different clinical–pathological characteristics and prognosis. For example, subtypes with significantly downregulated CLEC3B are more common in young, betel nut chewing female patients and have a high risk of recurrence [108,109]. Its anticancer mechanism is mainly attributed to the inhibition of the activity of urokinase-type plasminogen activator (PLAU) and its substrate fibronectin (FN1). Through this inhibition, the mechanism regulates extracellular matrix remodeling [108].

In thyroid follicular tumors, the downregulation of CLEC3B expression helps distinguish between benign and malignant properties [110]. In malignant follicular thyroid carcinoma (FTC), the expression of CLEC3B is significantly lower than in benign follicular thyroid adenoma (FTA), and its downregulation is associated with hypermethylation of the gene promoter. CLEC3B exerts a tumor-suppressive effect by maintaining the differentiated state of thyroid follicular cells and inhibiting pro-proliferative signaling pathways such as MAPK/ERK [110]. Its low expression indicates a higher risk of recurrence and metastasis.

In chronic lymphocytic leukemia (CLL), CLEC3B plays a tumor suppressive role, and its downregulation is closely associated with poor prognosis. As a key module gene identified by Weighted Gene Co-expression Network Analysis (WGCNA), low expression of CLEC3B is an independent indicator for predicting increased risk of disease recurrence and shortened overall survival [111,112]. The mechanism may involve regulating cellular processes such as ribosomal protein translation and affecting immune cell infiltration in the tumor microenvironment.

In osteosarcoma, CLEC3B also exhibits clear anticancer properties. Its expression in metastatic osteosarcoma tissue is significantly lower than that in non-metastatic tissue, and its low expression is associated with shorter metastatic survival and overall survival [113]. CLEC3B may reduce extracellular matrix degradation by promoting tumor cell apoptosis and inhibiting the plasminogen fibrinolytic system, thereby limiting tumor invasion and distant metastasis [113]. Therefore, it is a potential predictive biomarker for the risk of osteosarcoma metastasis.

In multiple myeloma (MM), the role of CLEC3B is closely associated with anti-CD38 monoclonal antibody therapy (such as daratumumab) [114]. The treatment leads to an upregulation of CLEC3B expression in CD34^+^ hematopoietic stem/progenitor cells within the bone marrow. Highly expressed CLEC3B enhances cell adhesion, causing CD34^+^ cells to be more firmly retained in the bone marrow microenvironment, thereby significantly inhibiting the mobilization and collection efficiency of hematopoietic stem cells and delaying hematopoietic reconstitution after autologous stem cell transplantation [114,115]. Therefore, in the therapeutic context of MM, CLEC3B is a key molecule that mediates treatment-related complications and thus exerts a negative impact on therapeutic efficacy. 

In laryngeal squamous cell carcinoma (LSCC), CLEC3B exhibits a unique oncogenic role, driven by an intermittent hypoxic microenvironment [107,108,109]. Under these conditions, M2 tumor-associated macrophages were significantly induced and highly secreted CLEC3B. This protein upregulates the expression of the key glycolytic enzyme hexokinase 1 (HK1) by activating the “CLEC3B-ZBTB10-HK1” signaling axis, thereby promoting the reprogramming of glycolytic metabolism in tumor cells, providing energy for rapid cell proliferation and migration, and ultimately enhancing the malignant progression of LSCC [109,116]. This mechanism reveals the special function of CLEC3B as a pro-cancer factor in the hypoxic microenvironment [107].

CLEC3B exhibits stromal-derived oncogenic functions in the pathological process of colorectal cancer (CRC). Unlike the tumor-intrinsic suppressive role observed in HCC or LUAD, CRC-associated CLEC3B is predominantly secreted by cancer-associated fibroblasts (CAFs), CRC-associated CLEC3B is predominantly secreted by cancer-associated fibroblasts (CAFs) and acts in a paracrine manner [117]. Its core function does not originate from the tumor cells themselves but rather from cancer-associated fibroblasts (CAFs). In the tumor microenvironment of CRC, CAFs are highly activated and secrete large amounts of CLEC3B, leading to its specific high expression within the tumor stroma. This CAF-derived CLEC3B acts as a key signaling molecule acting on CRC cells in a paracrine manner. Studies have shown that CLEC3B significantly enhances the migratory capacity of CRC cells such as SW480 and SW620, a mechanism involving the induction of cytoskeletal remodeling in tumor cells, including the promotion of expression of scaffold proteins such as F-actin and vinculin, as well as the formation of more filopodia. This process may be mediated by pathways such as Rho GTPase [117,118]. Clinical association analyses indicate that, particularly in cases of tumor serosal invasion, the co-expression of CLEC3B with the CAF marker α-SMA is significantly associated with poor patient prognosis. Additionally, CLEC3B expression exhibits age-dependent patterns, with overall expression levels declining progressively from healthy children to adults, and then to adenomas and CRC, suggesting that its expression is subject to age-related epigenetic regulation. In conclusion, in colorectal cancer, CLEC3B acts as a CAF-derived cancer-promoting factor, facilitating tumor cell migration by remodeling the tumor cytoskeleton [117]. In CRC, it is a key molecule that mediates the stromal-tumor cell interaction and drives tumor progression.

The dual roles of CLEC3B in tumors may depend on its cellular origin (tumor cell-derived versus stromal cell-derived), extracellular matrix composition, local proteolytic activity, and tumor type–specific signaling contexts, and the underlying mechanisms remain to be further investigated. The expression and function of CLEC3B in various tumor types are summarized (Table 1).

### 4.5. CLEC3B in Cardiovascular Diseases (CVDs)

In recent years, with the deepening of research on the pathological mechanism of CVD [119], the specific mechanism of action of CLEC3B in heart failure (HF) [119], coronary artery disease (CAD), and other diseases has gradually become clear [120]. It is not only a biomarker reflecting disease progression but also participates in the maintenance of cardiovascular homeostasis and pathological damage repair through multiple pathways, providing new directions for disease diagnosis, prognosis evaluation, and potential intervention targets.

CLEC3B has been implicated in the occurrence and progression of cardiovascular diseases. Based primarily on preclinical and observational evidence, three core pathways have been proposed to mediate this effect: regulation of fibrinolytic balance, inhibition of pathological fibrosis, and maintenance of endothelial function.Firstly, regulating plasminogen activation to maintain the balance of the coagulation–fibrinolysis system. As a specific binding protein of plasminogen, CLEC3B directly binds to the kringle 4 domain of plasminogen through its C-type lectin domain, forming a CLEC3B-plasminogen complex, which significantly enhances the activation efficiency of plasminogen [119]. Specifically, this complex amplifies the activation of plasminogen by tPA, accelerating the production of plasmin. As a key protease, plasmin rapidly degrades fibrin clots deposited in blood vessels, preventing thrombosis. In CAD and myocardial infarction (MI), this mechanism holds significant pathological importance: when atherosclerotic plaques rupture in coronary arteries, local activation of the coagulation system facilitates thrombus formation, leading to vascular occlusion. If CLEC3B levels are reduced, the efficiency of plasminogen activation decreases, and the fibrinolysis system fails to dissolve thrombi promptly, exacerbating myocardial ischemic injury and increasing the risk of MI and postoperative reinfarction. Conversely, adequate CLEC3B levels enhance fibrinolysis, reduce the duration of thrombotic vascular occlusion, mitigate myocardial cell necrosis, and provide protective effects on the acute prognosis of MI patients.

The second aspect is to inhibit pathological fibrosis of the myocardium and vascular wall and delay the process of remodeling. Myocardial fibrosis is the core pathological feature of HF progression, while vascular wall fibrosis is an important trigger for CAD vascular sclerosis [120]. CLEC3B directly inhibits the excessive fibrosis process by regulating fibrosis-related gene pathways. Mechanistic studies have shown that CLEC3B can exert anti-fibrotic effects by downregulating the activity of the TGF-β1/Smad signaling pathway: TGF-β1 is a key cytokine that induces fibroblast activation and promotes collagen synthesis. In the myocardial tissue of HF patients, an increase in TGF-β1 levels leads to the transformation of fibroblasts into myofibroblasts, which secrete a large amount of type I and III collagen, causing collagen deposition in the myocardial interstitium and affecting myocardial relaxation and contraction function. CLEC3B can competitively bind to the receptor-binding domain of TGF-β1, inhibiting the interaction between TGF-β1 and the receptor, thereby blocking Smad2/3 phosphorylation and nuclear translocation, reducing the transcription and expression of collagen genes (such as *COL1A1* and *COL3A1*), and alleviating myocardial interstitial fibrosis [119]. At the same time, in the context of vascular wall damage (such as the repair process of atherosclerotic plaque in CAD patients), CLEC3B can inhibit the abnormal proliferation and migration of vascular smooth muscle cells (VSMC). CLEC3B can downregulate the expression of cyclin D1 by binding integrin αvβ3 on the surface of VSMC, block VSMC in the G0/G1 phase, reduce its migration into plaque, and inhibit its phenotypic transformation into synthetic cells, preventing vascular wall thickening and lumen stenosis aggravation and delaying the progression of CAD [120].

Third, maintain vascular endothelial function and inhibit the onset of atherosclerosis [20]. Vascular endothelial dysfunction is an early sign of atherosclerosis. CLEC3B maintains endothelial homeostasis in two ways. One of them is to promote the survival and repair of endothelial cells (EC). CLEC3B can activate the phosphatidylinositol 3-kinase (PI3K)/protein kinase B (Akt) signaling pathway, upregulate the phosphorylation level of endothelial nitric oxide synthase (eNOS), and increase the production of nitric oxide (NO). NO, as a vasodilator factor, improves the anti-apoptotic ability of endothelial cells, reduces oxidative stress damage (such as inhibiting the generation of reactive oxygen species), and promotes the proliferation and migration of damaged endothelial cells, accelerating endothelial barrier repair [121]. Second, it inhibits the endothelial inflammatory response. Induced by risk factors such as hyperlipidemia, endothelial cells are prone to express adhesion molecules (such as ICAM-1, VCAM-1), recruit monocytes to adhere to and migrate into the vascular wall, and start the process of atherosclerosis [120]. CLEC3B can inhibit the initiation of atherosclerosis by inhibiting the activity of the nuclear factor-κB (NF-κB) pathway, reducing the transcription and expression of adhesion molecules, and reducing the binding efficiency of monocytes to endothelial cells.

Based on the above mechanism, multiple clinical studies have confirmed the clinical value of CLEC3B in the diagnosis and prognosis evaluation of cardiovascular diseases, specifically reflected in the following aspects. First, in the field of CAD diagnosis, CLEC3B helps to improve the accuracy of conventional cardiac biomarkers. A systematic review covering January 2010 to June 2025 (involving 12 observational studies in adults) demonstrated that CLEC3B exhibits specific expression patterns across different cardiovascular diseases. Circulatory CLEC3B levels in CAD patients (particularly those with stable angina or acute coronary syndrome) are 30%–40% lower than in healthy individuals (Figure 4) [122]. Moreover, CLEC3B levels within 24 hours of acute MI onset are negatively correlated with myocardial troponin I (cTnI)—lower CLEC3B levels indicate a larger extent of myocardial ischemic injury (Figure 4) [20]. Circulatory CLEC3B levels in patients with advanced HF (NYHA classes III–IV) are significantly lower than in early-stage patients (NYHA classes I–II) and are positively correlated with left ventricular ejection fraction (LVEF) (r = 0.42, *p* < 0.01), serving as a supplementary indicator for assessing HF severity (Figure 4). More importantly, two independent studies confirmed that the ‘combined detection strategy’—which involves co-testing CLEC3B with the traditional marker N-terminal brain natriuretic peptide precursor (NT-proBNP)—improved the area under the curve (AUC) for diagnosing HF from 0.78 to 0.89 when used alone, and increased the AUC for diagnosing CAD from 0.72 to 0.85 [20]. This approach effectively reduces false-positive/false-negative issues with NT-proBNP in obese and renal insufficiency patients, thereby enhancing diagnostic accuracy. Second, CLEC3B also contributes to CAD prognosis assessment, predicting the risk of adverse cardiovascular events. Long-term follow-up data indicate that circulating CLEC3B levels are independent predictors of cardiovascular disease prognosis. In patients with HF, those with CLEC3B levels ≥ 15 μg/mL had a 58% lower risk of cardiovascular death within 1 year compared to those with <10 μg/mL (HR = 0.42,95% CI: 0.28–0.63), and a 45% lower risk of rehospitalization (HR = 0.55,95% CI: 0.39–0.78) [110]. In patients with CAD, those with persistently low CLEC3B levels (<12 μg/mL) at 3 months postoperatively had a 2.3-fold higher risk of in-stent restenosis within 5 years compared to those with normal levels (OR = 3.3, 95% CI: 1.8–6.1). These results suggest that CLEC3B can indirectly predict disease progression risk by reflecting fibrinolytic function, fibrosis extent, and endothelial status, providing a basis for clinical risk stratification. In summary, CLEC3B has protective effects in preclinical models of cardiovascular diseases (particularly HF and CAD) through three core mechanisms: regulating fibrinolytic balance, anti-fibrotic effects, and maintaining endothelial function. It not only serves as a potential biomarker for disease diagnosis and prognosis assessment but also holds promise as a novel therapeutic target for cardiovascular diseases, providing preliminary theoretical foundations and potential clinical insights for precision prevention and treatment, pending prospective clinical validation.

### 4.6. The Role of CLEC3B in Eye-Related Diseases

**Figure 5:**
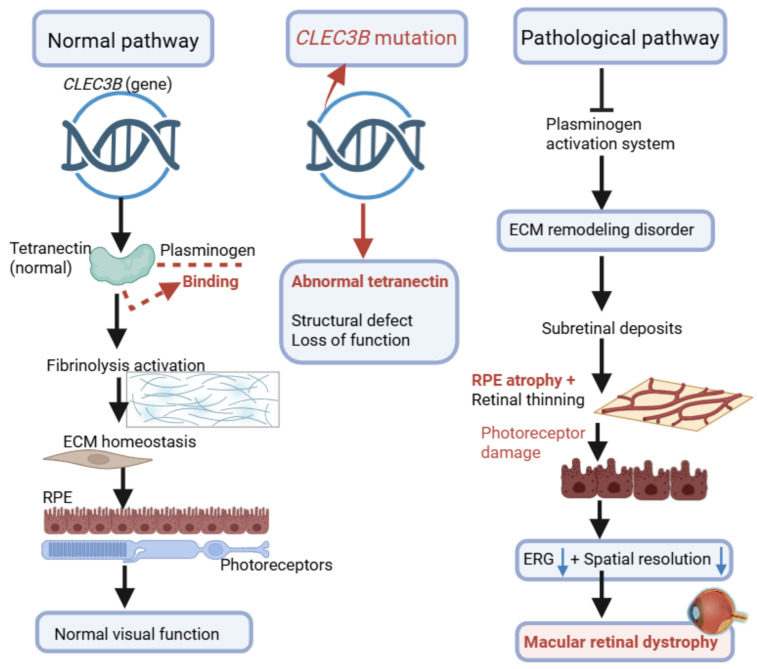
Schematic model of CLEC3B mutation-induced macular retinal dystrophy. Under normal conditions, the CLEC3B gene encodes functional tetranectin protein, which binds plasminogen and activates fibrinolysis, maintaining extracellular matrix (ECM) homeostasis in the retinal microenvironment. This homeostatic regulation supports the structural and functional integrity of the retinal pigment epithelium (RPE) and photoreceptors, ensuring normal visual function. Disease-associated mutations in CLEC3B (e.g., missense variants such as A180D) lead to the production of abnormal tetranectin with a structural defect, resulting in loss of normal protein function. This functional deficiency disrupts the plasminogen activation system and impairs ECM homeostasis within the subretinal space, leading to an ECM remodeling disorder. This promotes the progressive accumulation of subretinal hyper-reflective deposits. Subsequently, RPE atrophy and retinal thinning develop, leading to progressive photoreceptor damage. These pathological changes culminate in diminished electroretinographic (ERG) responses and impaired spatial resolution, ultimately manifesting as macular retinal dystrophy. Blue downward arrows indicate downregulation or inhibition. https://BioRender.com/qvr5uer (accessed on 21 June 2026).

Recent genetics studies have established a direct link between the CLEC3B mutation and inherited retinal disease [21], as shown in Figure 5. Through whole-exome sequencing and pathogenic variant analysis, CLEC3B was identified as a novel causative gene for autosomal dominant macular retinal dystrophy, a condition characterized by central vision loss due to photoreceptor and retinal pigment epithelium dysfunction.

This causal relationship was confirmed by functional modeling. Researchers used adeno-associated virus (AAV) to deliver a disease-associated human CLEC3B missense mutant (e.g., A180D) to the mouse subretinal space [21]. This intervention successfully recapitulated key features of the human disease, including the formation of subretinal hyper-reflective deposits, retinal thinning, and significantly diminished electroretinographic responses, confirming the pathogenicity of the variant in vivo [21]. Although the precise molecular mechanism by which mutant CLEC3B leads to retinal degeneration is still under investigation, its known functions provide plausible clues. Given CLEC3B’s role in regulating plasminogen activation and ECM homeostasis, it is hypothesized that the mutant protein may disrupt these processes in the highly specialized retinal microenvironment (Figure 5) [21]. This could result in abnormal protein aggregation, impaired clearance of toxic metabolites, or destabilization of the ECM supporting photoreceptors, ultimately triggering cell death and the observed clinical phenotype. Further studies are needed to dissect this pathogenic cascade.

In an exploratory serum proteomic analysis of patients with central serous chorioretinopathy (CSCR), levels of CLEC3B have been reported to potentially exhibit a decreasing trend, but this finding has not been adequately validated in independent, peer-reviewed cohort studies [123]. Existing histological evidence indicates that CLEC3B is predominantly expressed in the retinal pigment epithelium (RPE) and choroidal stroma of the human retina, and its potential role at the retinochoroidal interface remains to be further elucidated [2,8]. Although decreased levels of CELC3B have been observed in some exploratory studies, large-scale, independent, prospective cohort studies are lacking to validate its effectiveness and reliability as a biomarker for CSCR [123]. Therefore, the status of CLEC3B as a biomarker for CSCR still needs to be validated by more independent and high-quality studies.

The foregoing sections have delineated the pleiotropic roles of CLEC3B across diverse pathological contexts. Table 2 synthesizes these findings into a translational framework, summarizing CLEC3B’s biomarker utility (diagnostic, prognostic, and predictive), detection methodologies, and therapeutic rationale by disease category. All intervention strategies listed remain preclinical; no CLEC3B-targeted therapy has entered clinical trials.

## 5. Conclusions

CLEC3B, initially isolated from plasma, is now recognized as a multifunctional matrix-associated protein [125]. Its gene expression profile reveals a broad distribution with enrichment in tissues rich in secretory or endocrine cells [3]. As this review delineates, CLEC3B functions as a key regulator of ECM dynamics and cellular communication, integrating cues from the plasminogen activation system, growth factor signaling, and immune regulation. Its physiological roles—spanning tissue repair, bone mineralization, adipocyte differentiation, and neuronal survival—are indispensable for homeostasis [126]. Conversely, its dysregulation manifests across a remarkable spectrum of pathologies, including cancer, cardiovascular and neurodegenerative diseases, and ocular diseases such as inherited retinal dystrophy, often serving as a biomarker of disease severity and progression.

The pleiotropic nature of CLEC3B is underpinned by its structural capacity to engage diverse ligands (e.g., plasminogen, tPA, fibronectin, HGF) and its context-dependent signaling outcomes. This very complexity presents both a challenge and an opportunity for translational medicine. To date, CLEC3B has been proposed as a candidate biomarker and a preclinical therapeutic target across multiple disease categories; no CLEC3B-targeted intervention has yet entered clinical trials. The evidence-tier framework employed throughout this review provides a roadmap for prioritizing future investigations and distinguishing established biology from speculative hypotheses.

## 6. Future Perspectives

To bridge the gap between mechanistic understanding and clinical application, future research should prioritize the following directions:

Resolving the glycan-binding ambiguity. A fundamental unresolved question is whether CLEC3B retains any carbohydrate-recognition capacity. Given that its CTLD lacks canonical calcium-binding motifs yet preserves the overall lectin fold, systematic glycan microarray screening and high-resolution structural analysis of CLEC3B in complex with candidate sugar ligands should be prioritized. This will clarify whether CLEC3B is a true lectin with non-canonical glycan specificity or exclusively a protein-binding module—a distinction critical for both its molecular classification and therapeutic targeting.

Deciphering context-specific signaling networks. A major unanswered question is how CLEC3B elicits opposing effects (e.g., pro- vs. anti-tumor; pro- vs. anti-inflammatory) in different tissues or disease states. Systems biology approaches, single-cell omics, and spatially resolved proteomics in relevant disease models are needed to map the complete interactome and downstream pathways of CLEC3B, identifying the cofactors and microenvironmental conditions that determine its functional output.

Developing advanced disease models. Current CLEC3B-knockout mice, while invaluable, may not fully replicate human disease complexity or tissue-specific functions. The generation of conditional, cell-type-specific knockout or knock-in models (e.g., carrying the OA-associated S106G variant or retinal dystrophy mutants) will allow for precise dissection of their roles in tissue homeostasis maintenance, chronic diseases, and aging.

Translating biomarker potential into clinical tools. As detailed in Table 2, the biomarker utility of CLEC3B spans diagnostic (e.g., plasma CLEC3B for CAD), prognostic (e.g., CSF CLEC3B for PD), and predictive (e.g., CLEC3B-guided immunotherapy stratification in lung cancer) dimensions. The evaluative frameworks established in Section 1 provide the criteria underlying these applications. The consistent association of circulating CLEC3B levels with disease severity and prognosis across cancer, cardiovascular disease, and sepsis warrants large-scale, prospective multi-center studies to validate its utility as a standardized liquid biomarker. Simultaneously, exploring CLEC3B in exosomes or platelet releasates could open avenues for minimally invasive monitoring.

Exploring therapeutic targeting strategies. The dual role of CLEC3B as a pathophysiological mediator and a protective factor calls for nuanced therapeutic strategies. In conditions of deficiency (e.g., sepsis, neurodegenerative diseases), strategies to restore or supplement CLEC3B activity—using recombinant protein, gene therapy, or agonists of its production—hold promise. Conversely, in diseases driven by aberrant CLEC3B activity in specific local microenvironments —such as certain fibrotic or neoplastic contexts—targeted inhibition of its key interactions(e.g., using neutralizing antibodies or small-molecule disruptors) may offer therapeutic benefits. The CLEC3B–AMPK–HIF-1α axis and its hypothesized role in coupling angiogenesis with immune evasion represent a candidate target for combination therapy in cancer, pending functional validation in immunocompetent models.

## Figures and Tables

**Table 1:** Expression levels and roles of CLEC3B in different types of tumors.

Tumor Type	Expression Change	Functional Role	Key Mechanisms	Clinical Significance	References
Hepatocellular carcinoma (HCC)	Downregulated	Tumor suppressor	Its downregulation inhibits AMPK and upregulates VEGF to promote EMT and angiogenesis. It also promotes immune escape through the STAT3/PD-L1/MDSCs axis. CAF-derived TGF-β1 induces ECM loosening and targeted drug resistance by inhibiting CLEC3B to activate the PI3K/AKT pathway.	Its low expression predicts poor prognosis.	[74]
Lung cancer (LUAD/NSCLC)	Downregulated	Tumor suppressor	Its downregulation induces EMT, promotes inflammation, upregulates PD-L1, recruits M2 macrophages and MDSCs, and enhances tumor–CAF interaction.	Its low expression predicts poor prognosis.	[81,84]
Cholangiocarcinoma (CCA)	Downregulated	Tumor suppressor	It promotes β-catenin degradation by reducing GSK-3β phosphorylation and inhibits EMT.	Its low expression predicts poor prognosis.	[89]
Gallbladder cancer (GBC)	Downregulated	Tumor suppressor	It regulates cell adhesion, EMT, and ECM remodeling. Its overexpression inhibits proliferation and migration and induces apoptosis.	Its low expression predicts poor prognosis.	[90]
Pancreatic ductal adenocarcinoma (PDAC)	Downregulated	Tumor suppressor	It functions through three ways: direct tumor inhibition, remodeling of the immune microenvironment, and anti-angiogenesis.	Its low plasma level is used as an early biomarker. Its low expression predicts poor prognosis.	[91,92]
Clear cell renal cell carcinoma (ccRCC)	Downregulated	Tumor suppressor	It inhibits the phosphorylation of p38/ERK in the MAPK pathway.	Its low expression predicts poor prognosis.	[96]
Breast cancer (BC)	Downregulated	Tumor suppressor	It inhibits EMT, synergizes with SLIT2 to inhibit p38 MAPK/Erk signaling, and positively regulates immune TME.	Its low expression predicts poor prognosis.	[97]
Cervical cancer (CCC)	Downregulated	Tumor suppressor	Unknown (associated with bone metastasis)	Its low expression predicts poor prognosis.	[101]
Gastric cancer (GC)	Downregulated (systemic)	Tumor suppressor	Its downregulation promotes ECM degradation, angiogenesis, CAF activation, and anoikis resistance.	Its low expression predicts increased metastasis risk	[103]
Oral squamous cell carcinoma (OSCC)	Downregulated	Tumor suppressor	It inhibits plasminogen activation, reduces ECM degradation, and maintains an immune-activated TME.	Its low expression predicts poor prognosis.	[107]
Head and neck squamous cell carcinoma (HNSCC)	Downregulated	Tumor suppressor	Unknown	Its low expression predicts poor prognosis.	[108]
Follicular thyroid cancer (FTC)	Downregulated	Tumor suppressor	It inhibits the MAPK/ERK pathway.	Its low expression predicts higher metastasis risk and shorter survival.	[110]
Chronic lymphocytic leukemia (CLL)	Downregulated	Tumor suppressor	It affects ribosomal translation and the infiltration of immune cells into the tumor microenvironment.	Its low expression predicts higher recurrence risk and shorter overall survival (OS).	[111]
Osteosarcoma	Downregulated	Tumor suppressor	It promotes apoptosis and inhibits the plasminogen–fibrinolytic system, thereby reducing ECM degradation.	Its low expression predicts poor prognosis.	[113]
Multiple myeloma (MM)	Upregulated (post-therapy)	Therapy-limiting factor	It enhances adhesion and retention of CD34^+^ cells, inhibits mobilization efficiency and collection yield, and delays hematopoietic reconstitution after autologous stem cell transplantation (ASCT).	Its high expression impairs stem cell mobilization and delays hematopoietic reconstitution after ASCT.	[114]
Laryngeal squamous cell carcinoma (LSCC)	Downregulated (hypoxia)	Proto-oncogene	It upregulates HK1 through the CLEC3B–ZBTB10–HK1 axis and promotes glycolytic reprogramming.	Its high expression is associated with shorter disease-free survival (DFS) and OS.	[116]
Colorectal cancer (CRC)	Upregulated (tumor stroma)	Proto-oncogene	CAF-derived CLEC3B remodels the cytoskeleton and promotes migration.	Its high expression predicts a poor prognosis.	[117]

**Table 2:** Biomarker and therapeutic applications of CLEC3B.

Disease Category	Biomarker Application	Detection Methods	Therapeutic Target Rationale	Intervention Strategy
**Cancer**				
Hepatocellular carcinoma (HCC)	Prognostic biomarker [74]	Exosomal ELISA; tissue IHC; TCGA RNA-seq	(a) Downregulated in tumor (b) Overexpression inhibits EMT AMPK activators (c) Recombinant CLEC3B(rCLEC3B) protein deliverable	rCLEC3B supplementation restores tumor suppression.
Lung adenocarcinoma (LUAD)/NSCLC	Predictive biomarker [84]	TCGA database; clinical samples; immunohistochemistry	(a) High promoter methylation (b) Restoration inhibits EMT and immunosuppressive TME (c) Demethylating agents available	CLEC3B silencing by promoter hypermethylation; reactivated by demethylating agents.
Pancreatic ductal adenocarcinoma (PDAC)	Diagnostic biomarker [92,94]	IHC/PCR; plasma proteomics; mouse models	(a) Downregulated in tumor and metastases (b) Estrogen-responsive stromal expression (c) Exosome-mediated delivery is feasible	Intratumoral estrogen induces CLEC3B expression in stromal fibroblasts, creating an anti-tumor microenvironment.
Colorectal cancer (CRC)	Prognostic biomarker [4,117]	IHC; in vitro migration assays	(a) CAF-derived CLEC3B promotes migration (b) Paracrine oncogenic mechanism distinct from tumor-suppressive role in other cancers	Blocking CAF-derived CLEC3B or inhibiting CAF activation.
**Cardiovascular Diseases**				
Coronary artery disease (CAD)	Diagnostic biomarker [50]	Serum ELISA; meta-analysis of 12 cohort studies	(a) Circulating CLEC3B is 30–40% lower than in healthy controls (b) Combined with NT-proBNP improves AUC from 0.72 to 0.85	rCLEC3B restores fibrinolytic balance and endothelial function.
Heart failure (HF)	Prognostic biomarker [44,119]	Plasma samples were analyzed by ELISA in a pro-spective cohort with long-term follow-up	(a) Circulating CLEC3B levels correlate with LVEF (r = 0.42) (b) Levels ≥ 15 μg/mL associated with 58% lower cardiovascular death risk	CLEC3B supplementation inhibits cardiac fibrosis; TGF-β modulation attenuates remodeling.
Myocardial infarction (MI)	Diagnostic biomarker [119,121]	Serum ELISA; LC-MS/MS	(a) Negative correlation with cTnI (b) Reflects ischemic injury extent	Acute CLEC3B infusion promotes thrombolysis and ECM repair.
**Neurodegenerative Diseases**				
Parkinson’s disease (PD)	Diagnostic biomarker [46,61]	CSF proteomics; LC-MS/MS	(a) Knockout(KO) mice show PD-like phenotype (b) Exogenous CLEC3B inhibits p70S6K1 and neuronal apoptosis (c) Protein internalizable	Intrathecal injection of rCLEC3B or p70S6K1-targeted neuroprotective peptides.
**Musculoskeletal Diseases**				
Osteoarthritis (OA)	Diagnostic/risk biomarker [124]	GWAS genotyping; cartilage transcriptomics	(a) p.S106G missense variant linked to knee OA susceptibility (b) Upregulated in OA cartilage	Gene editing; small-molecule drugs that adjust how CLEC3B binds plasminogen, thereby controlling cartilage matrix turnover.
**Ocular Diseases**				
Macular retinal dystrophy	Diagnostic biomarker [21]	Whole-exome sequencing	(a) Monogenic disease with defined molecular mechanism (b) AAV-mediated gene delivery validated in vivo	AAV-mediated CLEC3B gene replacement therapy restores retinal ECM homeostasis.

## Data Availability

Not applicable.

## Abbreviations

The following abbreviations are used in this manuscript:

CLEC3B	C-type lectin domain family 3 member B
TN	Tetranectin
PD	Parkinson’s disease
CTLs	C-type lectins
CTLD	C-type lectin-like domain
CRD	Carbohydrate recognition domain
ECM	Extracellular matrix
FBN/FReD	Fibrinogen-like domain
LBS	Lysine-binding sites
tPA	Tissue plasminogen activator
Plg	Plasminogen
HGF	Hepatocyte growth factor
BSP	Bone sialoprotein
PBD	Plasminogen-binding domain
rmCLEC3B	Recombinant mouse CLEC3B
DAMP	Damage-associated molecular pattern
PON1	Paraoxonase 1
HDL	High-density lipoprotein
LDL	Low-density lipoprotein
AMPK	Adenosine 5′-monophosphate (AMP)-activated protein kinase
HIF-1α	Hypoxia-inducible factor-1α
VEGF	Vascular endothelial growth factor
CSF	Cerebrospinal fluid
p70S6K1	Protein S6 kinase beta-1
MN9D	Mouse mesencephalic dopaminergic neuronal cell line
OA	Osteoarthritis
hMSCs	Human mesenchymal stem cells
SMA	Spinal muscular atrophy
MS	Multiple Sclerosis
BBB	Blood–brain barrier
HCC	Hepatocellular carcinoma
CAL	Cancer-related lymphedema
TME	Tumor immune microenvironment
MDSCs	Myeloid suppressor cells
CAFs	Cancer-associated fibroblasts
LUAD	Lung adenocarcinoma
NSCLC	Non-small cell lung cancer
CCA	Cholangiocarcinoma
GBC	Gallbladder cancer
PDAC	Pancreatic ductal adenocarcinoma
ccRCC	Renal clear cell carcinoma
CNDE	Copy number deletion
VHL	von Hippel–Lindau tumor suppressor
BC	Breast cancer
CCC	Cervical cell cancer
TMB	Tumor mutation burden
GC	Gastric cancer
OSCC	Oral squamous cell carcinoma
HNSCC	Head and neck squamous cell carcinoma
PLAU	Urokinase type plasminogen activator
FTC	Follicular thyroid carcinoma
FTA	Follicular thyroid adenoma
CLL	Chronic lymphocytic leukemia
WGCNA	Weighted Gene Co-expression Network Analysis
MM	Multiple myeloma
LSCC	Laryngeal squamous cell carcinoma
HK1	Hexokinase 1
CRC	Colorectal cancer
CVDs	Cardiovascular diseases
HF	Heart failure
CAD	Coronary artery disease
MI	Myocardial infarction
VSMC	Vascular smooth muscle cells
EC	Endothelial cells
PI3K	Phosphatidylinositol 3-kinase
eNOS	Endothelial nitric oxide synthase
NO	Nitric oxide
NF-κB	Nuclear factor-κB
LVEF	Left ventricular ejection fraction
AUC	Area under the curve
AAV	Adeno-associated virus
CSCR	Central serous chorioretinopathy
RPE	Retinal pigment epithelium
